# Size-controlled bimodal *in vivo* nanoprobes as near-infrared phosphors and positive contrast agents for magnetic resonance imaging

**DOI:** 10.1080/14686996.2021.1887712

**Published:** 2021-03-10

**Authors:** Kyohei Okubo, Ryuta Takeda, Shuhei Murayama, Masakazu Umezawa, Masao Kamimura, Kensuke Osada, Ichio Aoki, Kohei Soga

**Affiliations:** a Department of Materials Science and Technology, Tokyo University of Science, Tokyo, Japan; b Group of Quantum-state Controlled MRI, National Institutes for Quantum and Radiological Science and Technology (QST), Chiba, Japan

**Keywords:** Bioimaging, magnetic resonance imaging, positive contrast agents, near-infrared fluorescence, rare-earth doped ceramics nanoparticles, 204 Optics / Optical applications, 302 Crystallization / Heat treatment / Crystal growth, 504 X-ray / Neutron diffraction and scattering, 505 Optical / Molecular spectroscopy

## Abstract

Rare-earth-doped nanoparticles (NPs), such as NaGdF_4_ nanocrystals doped with light-emitting rare earth ions, are promising bimodal probes that allow the integration of over 1000 nm near-infrared (OTN-NIR; NIR-II/III) fluorescence imaging and magnetic resonance imaging (MRI) of live bodies. A precise control of the particle size is the key factor for achieving a high signal-to-noise ratio in both NIR fluorescence and MR images and for regulating their function in the body. In this study, size-controlled NaGdF_4_:Yb^3+^, Er^3+^ NPs prepared by stepwise crystal growth were used for in vivo bimodal imaging. Hexagonal NaGdF_4_:Yb^3+^,Er^3+^ NPs coated with poly(ethylene glycol)-poly(acrylic acid) block copolymer, with hydrodynamic diameters of 15 and 45 nm, were prepared and evaluated as bimodal NPs for OTN-NIR fluorescence imaging and MRI. Their longitudinal (*T*_1_) and transverse (*T*_2_) relaxation rates at the static magnetic field strength of 1.0 T, as well as their cytotoxicity towards NIH3T3 cell lines, were evaluated and compared to study the effect of size. Using these particles, blood vessel visualization was achieved by MRI, with the highest relaxometric ratio (*r*_1_/*r*_2_) of 0.79 reported to date for NaGdF_4_-based nanoprobes (*r*_1_ = 19.78 mM^−1^ s^−1^), and by OTN-NIR fluorescence imaging. The results clearly demonstrate the potential of the size-controlled PEG-modified NaGdF_4_:Yb^3+^,Er^3+^ NPs as powerful ‘positive’ *T*_1_-weight contrast MRI agents and OTN-NIR fluorophores.

## Graphical abstract

**Figure:**
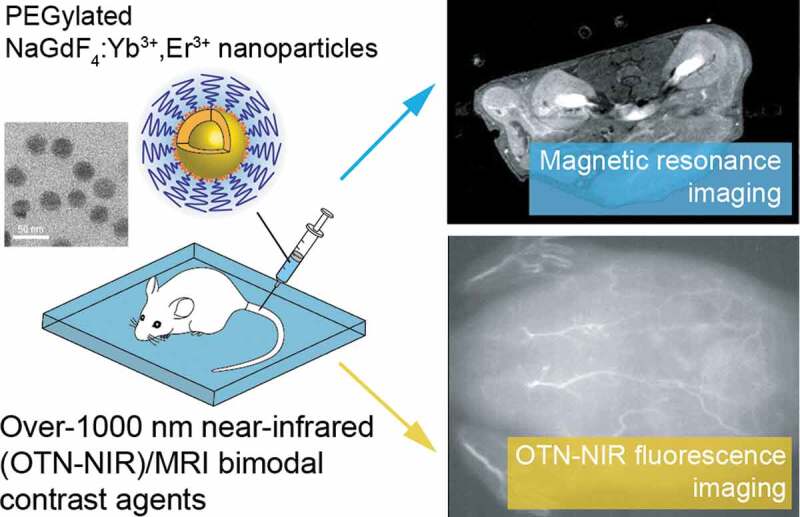

## 1. Introduction

Precise detection and diagnosis are key for the successful treatment of diverse diseases at an early stage [1,2]. Over the past decades, various imaging techniques, including fluorescence imaging, magnetic resonance imaging (MRI), X-ray computed tomography, positron emission tomography, and ultrasound, have been developed and combined for bimodal or multimodal imaging. These modalities have led to a rapid progress in understanding the pathophysiological phenomena in a live body [3–7]. Among these modalities, MRI is one of the foremost techniques used for developing the multimodal imaging technology because it facilitates deep tissue analysis and high soft-tissue contrast, and it is also widely used in clinical studies. To improve the spatial resolution of MR images, small paramagnetic molecules, such as Gd^3+^-based longitudinal (*T*_1_) contrast agents, are generally used for shortening the relaxation time of water, because Gd complexes are stable, and they maintain strong paramagnetic characteristics [8,9]. However, the spatiotemporal resolution and sensitivity provided by these NPs should be improved further for their practical application in tumor detection.

Optical imaging using fluorescent nanomaterials that can also enhance the MRI signal is considered to be a powerful strategy, owing to high spatiotemporal resolution and high sensitivity of the nanomaterials [10,11]. Near-infrared (NIR) fluorescence imaging has received increased attention in molecular imaging and clinical diagnosis owing to its advantages over imaging in the visible spectral range. The low absorption and scattering of the NIR light by biological tissues enables the investigation of deep tissues down to the depth of a few centimeters [12,13]. According to the optical properties of the target (i.e. skin, blood, or organs) and the availability of the imaging probes, the NIR ranges of NIR-I (650–950 nm), NIR-II (1000–1350 nm) [14,15], and NIR-III (1500–1800 nm) are classified as biological windows [16,17]. A window that combines NIR-II and NIR-III is also collectively referred to as over 1000 nm wavelength near-infrared (OTN-NIR) window [12,18]. Various OTN-NIR probes, including NIR organic dye-loaded polymer micelles [17], single-walled carbon nanotubes [19–22], Ag_2_S-based semiconductor quantum dots (QDs) [23,24], and rare-earth-doped ceramic NPs (RED-CNPs) [25,26], have been demonstrated to be applicable in the *in vivo* bioimaging of deep tissues. Among these OTN-NIR nanoprobes, RED-CNPs are promising multimodal agents owing to their narrow spectral lines, high photostability, low toxicity, and long NIR luminescence lifetimes [27,28]. In addition, their optical properties, including their absorption and fluorescence, can be precisely tuned by co-doping different rare-earth ions in the host matrix [12,16,29–31]. To ensure sufficient biocompatibility and dispersibility of the RED-CNPs in physiological environments, the surfaces of these NPs are often modified with biocompatible poly(ethylene glycol) (PEG)-based polymers. The PEG-modified RED-CNPs are not only suitable for visualizing blood vessels and organs in a live body, such as that of a live mouse [25,26], but also for nanothermometry [32–34] and photodynamic therapy [35–38].

From the perspective of the development of an OTN-NIR/MRI bimodal nanoprobe, Gd^3+^-containing NPs have been studied as bimodal or multimodal probes. Examples of such NPs include core–shell RED-CNPs [39–43] and Gd^3+^-doped iron oxide particles conjugated with fluorescence probes [44] or QDs [45]. The synthesis of core–shell RED-CNPs by layer-by-layer [46,47] or epitaxial growth [48] is a highly effective strategy for imparting them with multiple functions and controlling their size. A facile and reliable method for controlling the size of core–shell RED-CNPs is to add different amounts of the shell precursors (i.e. NaYF_4_ and NaGdF_4_) into the reaction vessel with a fixed amount of core RED-CNPs [49,50]. Although NaGdF_4_-based NPs, such as NaGdF_4_:Er^3+^,Yb^3+^ [39], NaYF_4_:Er^3+^,Yb^3+^@NaGdF_4_ [51], and NaErF_4_@NaGdF_4_@SiO_2_ [42] have been developed for upconversion fluorescence and MR bimodal imaging, only few studies have been reported on their use for NIR-II fluorescence imaging. Such studies include the use of 30 nm NaGdF_4_:Nd^3+^@NaGdF_4_ NPs for NIR-II/MRI bimodal liver cancer imaging [52] and the use of 50 nm NaYF_4_:5%Nd^3+^@NaGdF_4_ NPs for trimodal MRI, X-ray computed tomography, and OTN-NIR fluorescence imaging [53]. Further, most studies on the use of such core–shell NPs as contrast agents have focused on the demonstration of their multimodality and not on the evaluation of the intrinsic MR *T*_1_-weighted contrast using the ratio of the longitudinal (*r*_1_) to transverse relaxivity (*r*_2_), that is, *r*_1_/*r*_2_. In addition, the size of the RED-CNPs affects their luminescence properties and their capability to improve the MRI contrast; the NIR luminescence intensity of the RED-CNPs decreases significantly with a decrease in the NP size, and the relaxivities of the surrounding water protons are governed by the location of Gd^3+^ in the NPs and the NP size. Therefore, it is essential to develop efficient and robust methods that allow precise control over the size of the RED-CNPs for realizing optimal MRI contrast and fluorescence properties.

In this paper, we report the synthesis of size-controlled OTN-NIR/MRI bimodal contrast agents based on NaGdF_4_:Yb^3+^,Er^3+^ NPs coated with poly(ethylene glycol)-*block*-poly(acrylic acid) copolymer (PEG-*b*-PAAc). Specifically, we prepared PEGylated NaGdF_4_:Yb^3+^,Er^3+^ NPs with hydrodynamic diameters of 15 nm and 45 nm and evaluated their paramagnetic properties (*r*_1_ and *r*_2_), NIR fluorescence, and cell toxicity toward NIH3T3 cells. Further, we demonstrated *in vivo T*_1_-weighted MRI and OTN-NIR fluorescence imaging of the blood vessels of mice, which was realized by the intravenous administration of the PEG-NaGdF_4_:Yb^3+^,Er^3+^ NPs.

## 2. Experimental section

### 2.1. Materials

All reagents were used as received without further purification. PEG-*b*-PAAc (M_n_: 5000-*b*-2800 (g/mol), M_w_/M_n_: 1.2) was purchased from Polymer Source (Montreal, Canada). Oleic acid (OA, 90%) and nitrosyl tetrafluoroborate (NOBF_4_) were purchased from Sigma-Aldrich (St Louis, Missouri, US). Gadolinium(III) oxide (Gd_2_O_3_, 99.9%), ytterbium(III) oxide (Yb_2_O_3_, 99.9%), erbium(III) oxide (Er_2_O_3_, 99.99%), ethanol (99.5%), methanol (99.8%), 1-octadecene (ODE, 90.0%), cyclohexane (99.5%), trifluoroacetic acid (97.0%), sodium trifluoroacetic acid (CF_3_COONa, 98.0%), and *N,N*-dimethylformamide (DMF; 99.5%) were purchased from Wako Pure Chemical Industries (Osaka, Japan). Gadoterate meglumine (Gd-DOTA; Magnescope®) was purchased from Guerbet Japan, Inc. (Tokyo, Japan). Normal saline was purchased from Otsuka Pharmaceutical Factory, Inc. (Tokushima, Japan). Phosphate-buffered salt tablets were purchased from Takara Bio Inc. (Tokyo, Japan) to prepare phosphate-buffered saline (PBS).

### 2.2. Synthesis of PEGylated NaGdF_4_:Yb^3+^,Er^3+^ NPs

#### 2.2.1. Synthesis of OA-capped NaGdF_4_:Yb^3+^,Er^3+^ NPs

OA-capped NaGdF_4_:Yb^3+^,Er^3+^ NPs were prepared by a thermal decomposition method, as described previously [30,54]. For the synthesis of the precursor, 1.25 mmol of a mixture of rare earth oxides composed of 0.975 mmol of Gd_2_O_3_, 0.25 mmol of Yb_2_O_3_, and 0.025 mmol of Er_2_O_3_ (Gd:Yb:Er = 78:20:2 mol%) was mixed with 5 mL of distilled water and 5 mL of trifluoroacetic acid in a 100 mL three-necked round-bottom flask. Then, the mixture was heated at 130°C on an oil bath under magnetic stirring until a transparent solution was obtained. During this process, residual water and trifluoroacetic acid evaporated, and a rare earth–trifluoroacetic acid complex was obtained as a solid. For the synthesis of NaGdF_4_ NPs co-doped with 2 mol% Er^3+^ and 20 mol% Yb^3+^, the obtained precursor solid was dissolved in a solvent mixture comprising 2.5 mmol of trifluoroacetic acid sodium and 7.5 mL of ODE and pre-heated to 110°C in an oil bath. Subsequently, 7.5 ml of OA was added to the reaction mixture, and the resulting mixture was degassed for ~30 min at 130°C under vacuum. Another reaction vessel containing 12.5 mL of degassed ODE and 12.5 mL of OA was heated to 315°C for removing residual water. The hot precursor solution was then added to the reaction vessel using a syringe and pump system at an injection rate of 1.5 mL/min under nitrogen atmosphere. Next, the reaction mixture was cooled to room temperature (25°C). The OA-capped NaGdF_4_:Yb^3+^,Er^3+^ NPs were purified by centrifugal washing (2.0 × 10^4^ *g*, 10 min × 3) with ethanol and hexane and redispersed in 10 mL of cyclohexane.

#### 2.2.2. Stepwise crystal growth of NaGdF_4_:Yb^3+^,Er^3+^ NPs

The procedure for the stepwise crystal growth on the NP surfaces is based on that reported by Skripka et al. [55] NaGdF_4_:Yb^3+^,Er^3+^ NPs dispersed in 10 mL of cyclohexane were used as the precursors. To regulate the rate of the crystal growth around the NaGdF_4_ NPs, the procedure described above was applied, but the amount of the NaGdF_4_ NPs was changed with respect to that of the precursor solution, which was maintained constant. First, different amounts (20, 40, 60, 80, and 100 mg) of the NaGdF_4_:Yb^3+^,Er^3+^ NPs were added to the reaction vessel containing 12.5 mL of degassed ODE and 12.5 mL of OA, and the rare earth–trifluoroacetic acid complex solution was added to the vessel. Thereafter, the mixture was heated at 315°C for 45 min under nitrogen atmosphere.

#### 2.2.3. Preparation of PEG-NaGdF_4_:Yb^3+^,Er^3+^ NPs

The OA ligands on the surfaces of the NaGdF_4_:Yb^3+^,Er^3+^ NPs were replaced with PEG-*b*-PAAc by a stepwise ligand-exchange process, according to previously reported methods [17,56,57], with slight modifications. The OA-capped NaGdF_4_:Yb^3+^,Er^3+^ NPs were dispersed in cyclohexane (1 mg/mL) and 50 mg of NOBF_4_ was dissolved in 10 mL of DMF. Then, 5 mL of the OA-capped NaGdF_4_:Yb^3+^, Er^3+^ NPs dispersed in cyclohexane was gradually added to the DMF solution, and the mixture was stirred for 12 h at room temperature. To remove the aggregates in the DMF solution, the BF_4_-modified NaGdF_4_:Yb^3+^,Er^3+^ NPs were purified by centrifugal washing (1.0 × 10^4^ *g*, 10 min), and the purified DMF suspension of the BF_4_-NaGdF_4_:Yb^3+^,Er^3+^ NPs was further purified by centrifugal washing (4.0 × 10^4^ *g*, 20 min) with chloroform. The collected BF_4_-NaGdF_4_:Yb^3+^,Er^3+^ NPs were then redispersed in 10 mL of DMF. To replace the BF_4_ ligands with PEG-*b-*PAAc, the BF_4_-NaGdF_4_:Yb^3+^,Er^3+^ NP suspension was slowly added to a solution of PEG-*b*-PAAc (25 mg) in 5 mL of DMF. The mixture was stirred for 12 h, and the PEG-NaGdF_4_:Yb^3+^,Er^3+^ NPs were purified using dialysis membranes (Molecular weight cut-off: 15,000) through dialysis against water for 24 h. Finally, the PEG-NaGdF_4_:Yb^3+^,Er^3+^ NPs were dispersed in 5 mL of pure water.

### 2.3. Characterization of NaGdF_4_:Yb^3+^,Er^3+^ NPs

The morphologies and size distributions of the PEG-NaGdF_4_:Yb^3+^,Er^3+^ NPs were investigated by transmission electron microscopy (TEM; HD-2300, Hitachi, Japan). The crystalline phase of the OA-capped NaGdF_4_:Yb^3+^,Er^3+^ NPs was identified by X-ray diffraction (XRD) (RINT-TTRIII, RIGAKU, Japan). The hydrodynamic diameters of the NPs were determined using a dynamic light scattering (DLS) particle size analyzer (LB-250, Horiba, Japan); the OA-capped NaGdF_4_:Yb^3+^,Er^3+^ NPs and PEG-NaGdF_4_:Yb^3+^,Er^3+^ NPs used for DLS were diluted with cyclohexane and water, respectively, to a particle concentration of 1.0 and 17.6 mg/mL, respectively. The Gd^3+^ concentration in each sample was determined from the weight of NaGdF_4_:Yb^3+^,Er^3+^ NPs, whose stoichiometry was confirmed by the XRD analysis. The NIR luminescence images of the PEG-NaGdF_4_:Yb^3+^,Er^3+^ NPs were acquired using a portable NIR fluorescence *in vivo* imaging system (SAI-1000, Shimadzu, Japan) [31]. The colloidal stability of the PEG-NaGdF_4_:Yb^3+^,Er^3+^ NPs in physiological saline and pure water was investigated by measuring their absorbance at 550 nm using an ultraviolet-visible spectrophotometer (V630, JASCO, Japan).

### 2.4. Cytotoxicity assay

The cytotoxicity of the as-prepared PEG-NaGdF_4_:Yb^3+^,Er^3+^ NPs was evaluated *in vitro* through an MTT assay using NIH3T3 cells (ATCC). The NIH3T3 cells were seeded at a density of 10^4^ cells/well in a 96-well plate and cultured in a minimum essential medium containing 10% fetal bovine serum and 1% penicillin–streptomycin at 37°C in a humidified atmosphere with 5% CO_2_. The cells were exposed to different concentrations of the PEG-NaGdF_4_:Yb^3+^,Er^3+^ NPs, ranging from 0.01 to 10 mM, for 24 h at 37°C. The cells were then washed with 9.6 mM PBS. A mixture of water-soluble tetrazolium salts, WST-8, and the culture medium was added to each well, and the cells were incubated for 30 min. The cell viability was evaluated by measuring the absorption of WST-8 at 450 nm in a microplate reader (Multiscan FC, Thermo Scientific, Massachusetts, US). The experiment was repeated five times.

### 2.5. In vitro MR properties

The longitudinal and transverse relaxation times (*T*_1_ and *T*_2_, respectively) at different Gd^3+^ concentrations ranging from 0 to 1 mM were determined with an MRI system at 23°C. The NaGdF_4_:Yb^3+^,Er^3+^ NPs were dispersed in water, and the MRI signals of the NP samples were acquired with a 1.0 T-MRI scanner (ICON, Bruker Biospin, Ettlingen, Germany) using a volume coil (35 mm inner diameter, transmission and reception, Aspect Imaging, Shoham, Israel). An aqueous solution of the contrast agent was placed in a polymerization chain reaction (PCR) tube cluster plate, and the PCR tube cluster plate was set at the center of the volume coil. The sample was maintained at room temperature (23.0 ± 0.5°C) using a gradient coil cooling system and air conditioners. Then, using the MRI scanner, horizontal single-slice *T*_1_-weighted and *T*_2_-weighted MR images were acquired with the following parameters: spin echo, repetition time (TR) = 400 ms, echo time (TE) = 10 ms, slice thickness = 2.0 mm, matrix = 256 × 256, field of view (FOV) = 38.4 × 38.4 mm, number of averages (NA) = 1, number of slices = 1. For calculating *T*_1_ and *r*_1_, horizontal single-slice inversion-recovery MRI was performed through rapid acquisition using the relaxation enhancement (RARE) sequence with the following parameters: TR = 10,000 ms; TE = 20 ms; inversion time = 52, 100, 200, 400, 800, 1600, 3200, and 6400 ms; matrix size = 128 × 128; FOV = 38.4 × 38.4 mm; slice thickness = 2.0 mm; RARE factor = 4; and NA = 1. For *T*_2_ and *r*_2_ calculation, horizontal single-slice multi-echo imaging was conducted using the following parameters: TR = 15,000 ms, TE = 12 to 768 ms (12 ms interval, 64 steps), number of echoes = 64, matrix size = 64 × 64; FOV = 38.4 × 38.4 mm; slice thickness = 3.0 mm; and NA = 1. The total acquisition time was 16 min. The *r*_2_ relaxivity maps were obtained by a non-linear least square fit analysis using the multi-echo imaging. Then, the *r*_1_ and *r*_2_ relaxivities of each CNP were calculated from the concentration-dependent plots of the measured *T*_1_ and *T*_2_ relaxation times using the following equation:

(1) $$1/T_{i}=\;1/{T_{i}}_{\left(0\right)}+r_{i}\left[Gd^{3+}\right]\;\left(i=\;1\;and\;2\right)$$

where 1/*T_i_*_(0)_ (s^−1^) denotes the longitudinal (*i* = 1) or transverse (*i* = 2) relaxation rate in the absence of paramagnetic species. [Gd^3+^] represents the concentration of Gd^3+^ in the NP suspension.

### 2.6. In vivo MRI

The animal care for MRI experiments was performed in accordance with the guidelines of the care and use of laboratory animals, as stated by the National Institutes for Quantum and Radiological Science and Technology (QST, Chiba, Japan) and approved by the committee for the animal experiments of QST. Male BALB/c nu/nu mice (10 weeks old, 23.8 g, Japan SLC, Japan) were inoculated subcutaneously with 100 μL of Colon-26 cells (1 × 10^6^ cells/mL), and the tumor growth was allowed until the tumor size reached 500 mm^3^ (~5 mm in diameter). The mice were then anesthetized with a 3% isoflurane solution; they were placed in prone position, and anesthesia was maintained at 1.5% for MRI scanning. During the experiment, warm air was flown over the animal to maintain its body temperature at 36.5°C. The subcutaneous tumor-bearing mice were intravenously injected with 15 ± 4 and 45 ± 20 nm PEG-NaGdF_4_:Yb^3+^,Er^3+^ NPs via the tail vein at the dose of 2 µmol of Gd^3+^. The signal intensities of the regions of interest (ROI) in the large vessel (blood) and muscle were analyzed using a software (Osirix, Pixmeo SARL, Switzerland), and the results were compared after normalization.

For the analysis of the subcutaneous tumors, 2D multi-slice *T*_1_-weighted MR images were obtained using a conventional SE sequence with the following parameters: TR = 400 ms, TE = 10 ms, matrix size = 256 × 256, FOV = 30.0 × 30.0 mm^2^, slice thickness = 1.0 mm, Fat-Sup = on, and NA = 4. Slice orientation was transverse. The images were obtained at the nominal voxel resolution of 156 × 156 × 1000 µm^3^. For *T*_2_-weighted MRI, a multi-slice multi-echo SE sequence (TR = 3000 ms, slice thickness = 1 mm, FOV = 30.0 × 30.0 mm, matrix = 256 × 256, slice orientation = transaxial, and NA = 1) was used with echo times ranging from 10 to 140 ms in steps of 10 ms.

### 2.7. In vivo OTN-NIR fluorescence imaging

The animal care and experiments were performed in accordance with the guidelines of the care and use of laboratory animals, as stated by the Tokyo University of Science. Four-week-old male ICR mice were purchased from Japan SLC, Inc., Japan. Before the imaging experiments, the mice were fed with the AIN-76A diet (Research Diets Inc., New Jersey, US) for 2 weeks to reduce the levels of body phosphorescent alfalfa. Then, the mice (6 weeks old, 32.0 g) were anesthetized, and their hair was removed to eliminate light scattering. Subsequently, 200 µL of the PEG-NaGdF4:Yb^3+^,Er^3+^ NP suspension in normal saline (9 mg/mL sodium chloride) was injected into the tail vein at an injection rate of 13.3 µL/s. The NIR fluorescence images were obtained using an SAI-1000 system.

## 3. Results and discussion

### 3.1. Synthesis and characterization of size-controlled NaGdF_4_:Yb^3+^,Er^3+^ NPs

**Figure 1.**
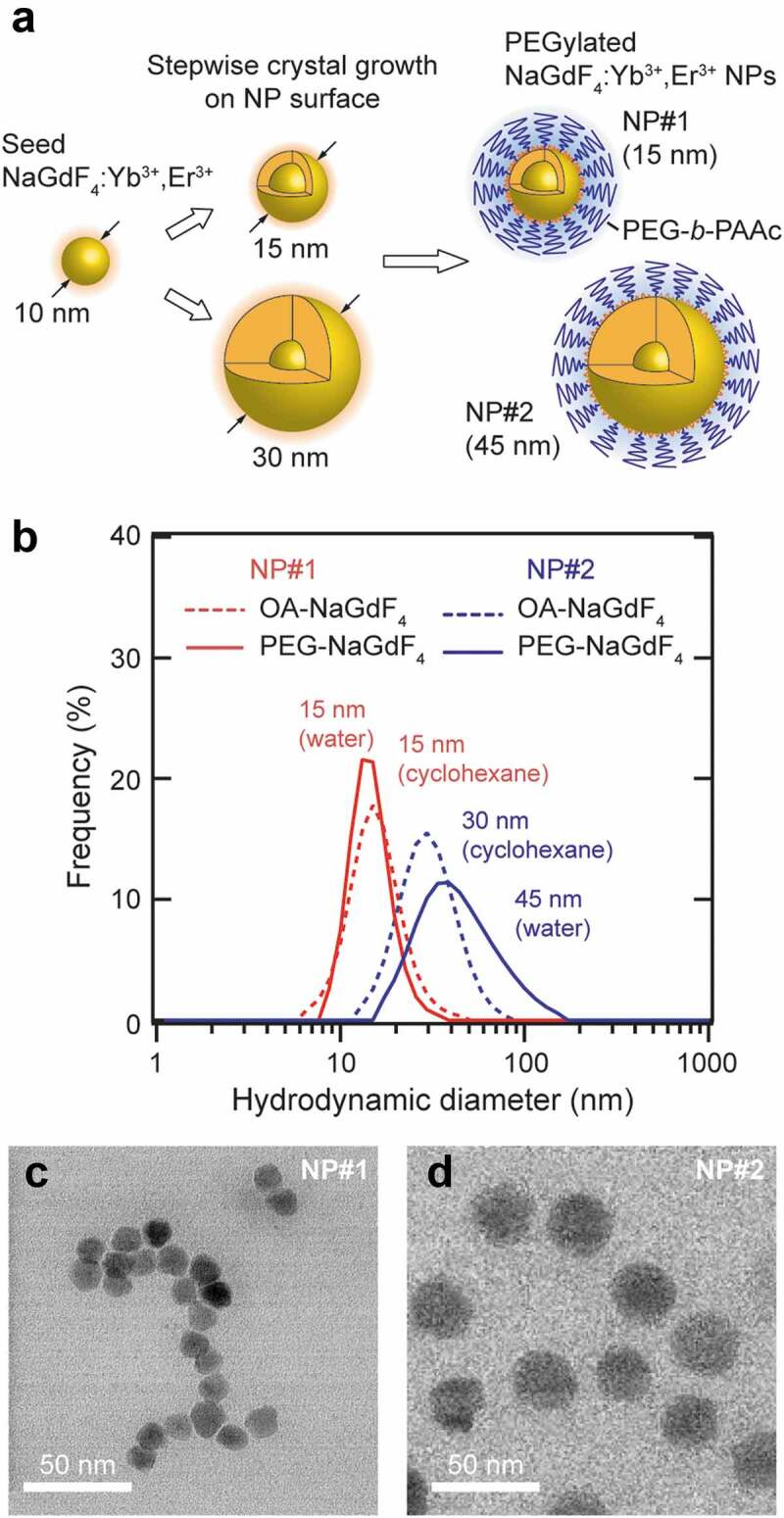
(a) Schematic illustration of the size-controlled synthesis of NaGdF_4_:Yb^3+^,Er^3+^ NPs by the stepwise crystal growth. (b) Particle size distribution of PEGylated and OA-capped NaGdF_4_:Yb^3+^,Er^3+^ NPs (DLS measurement conditions: sample concentration of 17.6 and 1.0 mg/mL, respectively, in distilled water and cyclohexane). TEM images of (c) 15 nm (NP#1) and (d) 45 nm (NP#2) PEG-NaGdF_4_:Yb^3+^,Er^3+^ NPs

In this study, size-controlled NPs were synthesized by the stepwise crystal growth around the seed NPs. The growth rate of the RED crystalline layer can be decreased by increasing the amount of the seed NPs in the reaction mixture, assuming that the crystalline layer grows uniformly on the surface of each particle. Therefore, the number density of the seed NPs in the suspension determines the thickness of the newly grown crystalline layer. Figure 1(a) displays a schematic of the synthesis process. First, the OA-capped NaGdF_4_:Yb^3+^,Er^3+^ NPs were synthesized by a thermal decomposition method from the rare earth ion–trifluoroacetate complex. In this study, the size of the seed NPs was controlled at 10 nm (Figure S1) using OA as the capping agent; OA also serves as a solvent in the reaction. Then, the crystalline layer composed of NaGdF_4_:Yb^3+^,Er^3+^ was grown around the seed NPs. The resultant NaGdF_4_:Yb^3+^,Er^3+^ NPs were modified with PEG-*b*-PAAc in DMF. Finally, the obtained PEG-NaGdF_4_:Yb^3+^,Er^3+^ NPs were characterized, and their properties as OTN-NIR/MRI bimodal nanoprobes were evaluated.

The sizes of the NaGdF_4_:Yb^3+^,Er^3+^ NPs (Yb^3+^:Er^3+^ = 20:2 mol%) prepared using 80 and 40 mg of the 10 nm NaGdF_4_:Yb^3+^,Er^3+^ NPs (referred to as NP#1 and NP#2, respectively) were determined by DLS in cyclohexane. Both these samples have a unimodal size distribution with a diameter of 15 ± 5 and 30 ± 20 nm, respectively (Figure 1(b)). Subsequent measurements with NP#1 modified with PEG-*b*-PAAc in water yielded a similar size as that of the original sample (Table 1). The Fourier-transform infrared spectra of the PEG-*b*-PAAc-modified CNPs exhibit peaks at 1116, 1730, and 2895 cm^−1^ corresponding to the C–O–C bond of PEG, –COOH group of PAAc, and C–H bonds of PEG and PAAc, respectively, indicating the successful PEGylation of the CNPs (Figure S2). Remarkably, the specific gravity of the CNPs, 5.612 g/cm^3^ for hexagonal NaGdF_4_ nanocrystals [58], is substantially higher than that of the flanking PEG chains. Therefore, it is likely that the diffusion behavior of the PEGylated CNPs, which is an essential feature for the DLS measurement, is mostly dominated by the CNPs themselves. In addition, PEG has a negligible impact on the size determination, leading to the same size of the NP determined by DLS even with the attachment of PEG. In contrast, NP#2 showed a size increase (45 ± 20 nm) and widening of the size distribution after its PEGylation (Figure 1(b)). We assume that the increased size is due to the aggregation of a few CNPs during the PEGylation process. The 1.5-time larger DLS size allowed the estimation of an aggregation number of 3.4, because the third power of 1.5 corresponds to 3.4. The TEM images of PEGylated NP#1 and NP#2 confirm the formation of particles with average sizes of 14 ± 1 and 28 ± 2 nm, respectively (*n* = 30) (Figure 1(c,d)). These sizes are consistent with the sizes determined by DLS. Note that only CNPs are visualized in the TEM images, whereas PEG is invisible due to its considerably lower electron density.

**Table 1.** Comparison of the longitudinal relaxivity (*r*_1_), transverse relaxivity (*r*_2_), and *r*_1_/*r*_2_ ratio of NaGdF_4_-based NPs. Note: PEG-*b*-PAAc, poly(ethylene glycol) poly(acrylic acid) block copolymer; PVP, polyvinylpyrrolidone; DSPE-PEG, 2-distearoyl-sn-glycero-3-phosphoethanolamine-N- [methoxy(polyethyleneglycol)-2000]

Nanoprobe	Hydrodynamic diameter (nm)	Surface coating	*r*_1_ (mM^−1^s^−1^)	*r*_2_ (mM^−1^s^−1^)	*r*_1_/*r*_2_	field (T)	references
β-NaGdF_4_:Yb^3+^,Er^3+^ (NP#1)	15	PEG-*b*-PAAc	14.46	19.43	0.744	1.0	this study
β-NaGdF_4_:Yb^3+^,Er^3+^ (NP#2)	45	PEG-*b*-PAAc	19.78	24.92	0.794	1.0	this study
Gd-DOTA	–	–	3.44	4.82	0.714	1.0	this study
β-NaGdF_4_	10	PVP	7.20	22.70	0.317	1.5	[59]
α-NaGdF_4_	28	PAAc	4.13	6.48	0.637	3.0	[60]
β-NaGdF_4_	27	PAAc	2.78	6.98	0.398	3.0	[60]
α-NaY(Gd)F_4_:Tm^3+^,Yb^3+^	6	citrate	3.37	3.96	0.851	1.4	[61]
β-NaY(Gd)F_4_:Tm^3+^,Yb^3+^	24	citrate	6.33	26.16	0.242	1.4	[61]
α-NaGdF_4_	16	DSPE-PEG	8.93	26.59	0.336	3.0	[62]
β-NaGdF_4_:Dy^3+^	–	DSPE-PEG	5.17	10.64	0.486	7.0	[63]
β-NaGdF_4_:Nd^3+^@NaGdF_4_ core–shell	31	liposome	0.25	2.17	0.114	3.0	[52]
β-NaGdF_4_:Nd^3+^@NaGdF_4_ core–shell	49	DSPE-PEG	0.89	–	–	0.5	[53]
β-NaGdF_4_:Yb^3+^,Er^3+^	10	octylamine-PAAc	0.99	–	–	4.7	[64]

**Figure 2.**
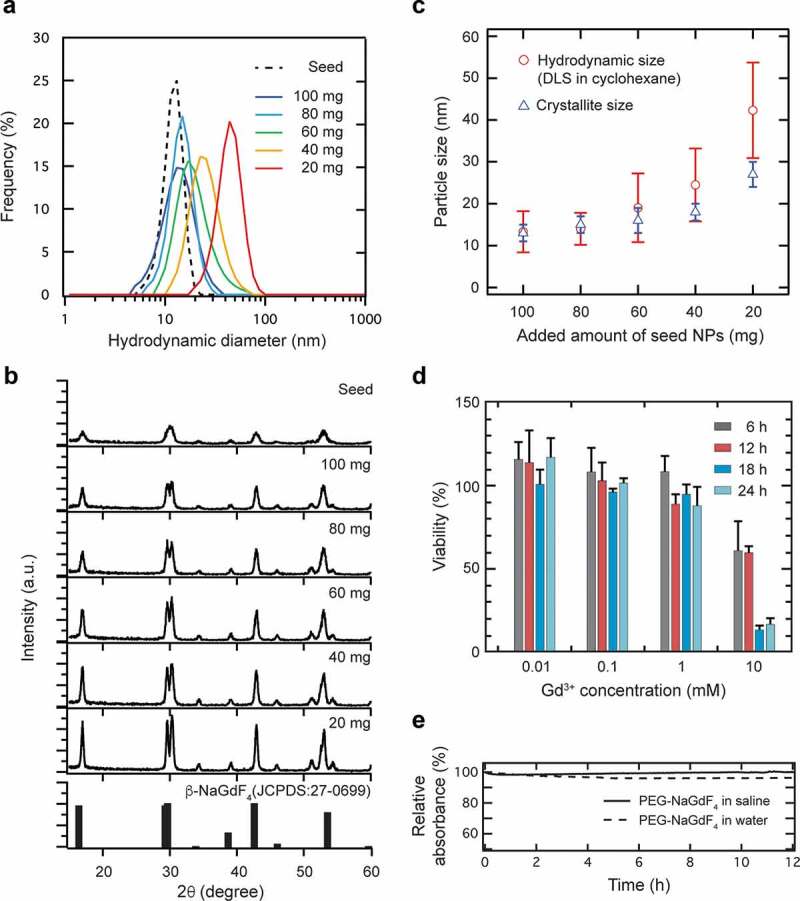
Particle size control based on the amount of seed NaGdF_4_:Yb^3+^,Er^3+^ NPs used in the reaction. (a) Particle size distribution of OA-NaGdF_4_:Yb^3+^,Er^3+^ NPs obtained with different amounts of seed NPs (20–100 mg), as determined by DLS (particle concentration: 1.0 mg/mL in cyclohexane). (b) XRD patterns of OA-NaGdF_4_:Yb^3+^,Er^3+^ NPs along with that of the reference sample, hexagonal β-NaGdF_4_. (c) Dependence of the particle size of NaGdF_4_:Yb^3+^,Er^3+^ NPs on the amount of added seed NPs. Crystallite sizes and hydrodynamic sizes were measured by XRD and DLS, respectively. (d) Cytotoxicity of the PEG-NaGdF_4_:Yb^3+^,Er^3+^ NPs (NP#1) against NIH3T3 cells, as determined by the MMT assay. NIH3T3 cells were exposed to different PEG-NaGdF_4_:Yb^3+^,Er^3+^ NPs with different Gd^3+^ concentrations (0.01, 0.1, 1, and 10 mM) for 6, 12, 18, and 24 h. Data are expressed as mean ± standard deviation (*n* = 5 for each sample). (e) Colloidal stability of 15 nm PEG-NaGdF_4_:Yb^3+^,Er^3+^ NPs in saline and deionized water, as evaluated by their absorbance at 550 nm

To examine the precise size-controllability of the NaGdF_4_:Yb^3+^,Er^3+^ NPs obtained by the stepwise crystal growth, the amount of the seed NPs added for the subsequent crystal growth was varied in the range of 20–100 mg. The DLS analysis indicated that the size of the synthesized NPs increased with a decrease in the amount of the added NPs (Figure 2(a)). The crystallite size can be estimated from the XRD pattern based on Scherrer’s equation, which correlates the size of the submicron crystallites to the full-width at half-maximum of the XRD peak. Note that the powder XRD studies revealed that the NaGdF_4_:Yb^3+^,Er^3+^ NPs were formed in the hexagonal β-phase of NaGdF_4_, and all the peaks corresponded with the JCPDS data for this phase (27–0699) (Figure 2(b)). The calculated crystallite size increased with a decrease in the amount of the added seed NPs, conforming with the hydrodynamic sizes measured by DLS (Figure 2(c)). Notably, the XRD peaks were broadened for CNPs prepared with a lesser amount of the added seed NPs, which may be attributed to the formation of some smaller crystallites. These evaluations suggest that the stepwise crystal growth method allowed the precise size-control of the NaGdF_4_:Yb^3+^,Er^3+^ NPs in the range of 10–30 nm.

Low cytotoxicity is an essential requirement for probes used in diagnostic applications. The viability of NIH3T3 cells was investigated *in vitro* using the standard MTT assay after 6, 12, 18, and 24 h of incubation of NP#1. None of the PEG-NaGdF_4_:Yb^3+^,Er^3+^ NPs showed apparent cytotoxicity in the range of 0.01–1 mM Gd^3+^ concentration (Figure 2(d)). In contrast, a significant decrease in the cell viability was observed at 10 mM Gd^3+^ concentration. Therefore, we conclude that the PEG-NaGdF_4_:Yb^3+^,Er^3+^ NPs have minimal toxicity at concentrations lower than 1 mM.

As nanoprobes that simultaneously facilitate MRI contrast and fluorescence imaging *in vivo*, NPs developed for imaging should be stable while they circulate in the bloodstream. We evaluated the colloidal stability of the PEG-NaGdF_4_:Yb^3+^,Er^3+^ NPs in physiological saline and pure water by monitoring their absorbance at 550 nm. The near-constant absorbance of NP#1, that is, 15 nm PEG-NaGdF_4_:Yb^3+^,Er^3+^ NPs, over 12 h indicated their high colloidal stability without detectable precipitation (Figure 2(e)).

### 3.2. In vitro relaxivity measurement of PEG-NaGdF_4_:Yb^3+^,Er^3+^ NPs

**Figure 3.**
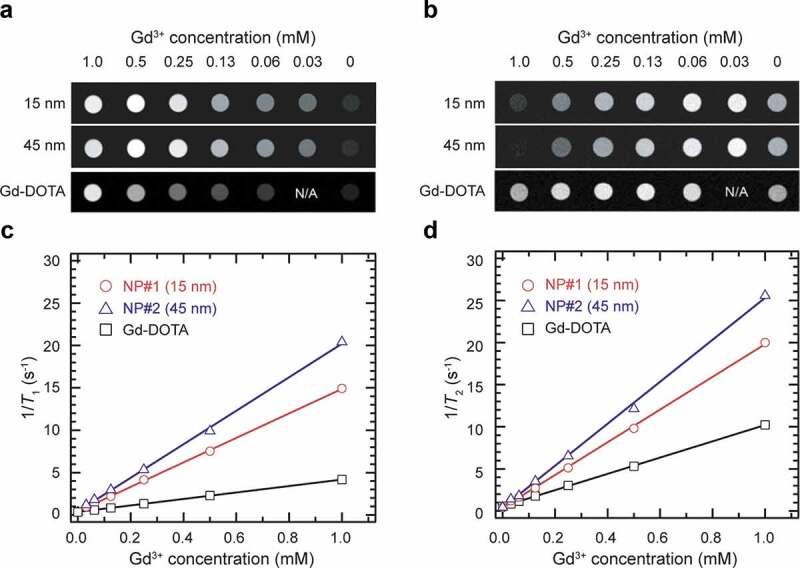
MR enhancement effects of 15 and 45 nm PEG-NaGdF_4_:Yb^3+^,Er^3+^ NPs. (a) *T*_1_-weighted and (b) *T*_2_-weighted MR images of the PEG-NaGdF_4_:Yb^3+^,Er^3+^ NP solution at the magnetic field strength of 1 T. (c, d) *T*_1_ and *T*_2_ relaxation rates of PEG-NaGdF_4_:Yb^3+^,Er^3+^ NPs determined with a 1.0 T-MRI scanner. *T*_1_ and *T*_2_ relaxation rates of Gd-DOTA, which was used as a reference, are also displayed in Figure 3(c,d)

The performance of the PEG-NaGdF_4_:Yb^3+^,Er^3+^ NPs as MRI contrast agents was evaluated using a preclinical 1.0 T MRI scanner at room temperature (23°C). The experimentally determined longitudinal and transverse relaxation rates (1/*T*_1_ and 1/*T*_2_) of water protons are plotted against the molar concentration of Gd^3+^, along with the results of Gd-DOTA used as a reference. Figure 3(a,b) show the *T*_1_-weighted and *T*_2_-weighted images obtained with different concentrations of NP#1, NP#2, and Gd-DOTA. Both NP#1 and NP#2 show higher *T*_1_ and *T*_2_ contrast enhancement than that of Gd-DOTA (Figure 3(c,d)). Whereas the *r*_1_ value of Gd-DOTA is 3.44 mM^−1^ s^−1^, the *r*_1_ value of NP#1 increased nearly four times to 14.46 mM^−1^ s^−1^ and that of NP#2 increased nearly six times to 19.78 mM^−1^ s^−1^.

The intrinsic ability of ‘positive’ *T*_1_ contrast agents to enhance an MR signal is dominated by the ratio of their longitudinal relaxivity (*r*_1_) to transverse relaxivity (*r*_2_), that is, the *r*_1_/*r*_2_ ratio. A high *r*_1_ with a low *r*_2_ provides high signal enhancement in the *T*_1_-weighted image, and thus a high *r*_1_/*r*_2_ ratio is considered to be ideal. For NaGdF_4_ NP-based probes, most of the previous reports only presented the *r*_1_ and omitted the *r*_2_, despite its major impact on their performance as a ‘positive’ contrast agent. The *r*_1_ values of the NP#1 and NP#2 are significantly higher than those reported for NaGdF_4_-based probes [52,53,59–64], as summarized in Table 1. Most importantly, the *r*_1_/*r*_2_ values of both NP#1 (0.744) and NP#2 (0.794) are higher than those of others, including Gd-DOTA, indicating that both NPs can be assumed to have suitable ‘positive’ contrast properties. The relaxivity is affected by multiple factors, including the difference in the applied magnetic field, accessibility of water to the particle surface, location of paramagnetic ions in the NP, and NP size. Among them, NP size is one of the important factors to be considered in the design of MRI–NIR bimodal probes. The contribution of the particle size to *r*_1_ varies with the size range of NPs. It has been reported that small particles (<10 nm in size) show high *r*_1_ owing to the high ratio of the surface-to-core ions [59,60], while Gd^3+^ at the surface of larger particles (>10 nm diameter) makes a larger contribution to *r*_1_/*r*_2_ than the surface Gd^3+^ on ultrasmall particles (3 nm diameter) [65]. However, the high *r*_1_/*r*_2_ ratios of NP#1 and NP#2 are due to lesser changes in *r*_2_ with an increase in the particle size. In the case of ferromagnetic contrast agents, such as iron oxides (e.g. Fe_2_O_3_), larger NPs show noticeably higher *r*_2_ values [66], because of which they show negative contrast enhancement. Paramagnetic NaGdF_4_ NPs, however, show relatively constant *r*_2_ values, regardless of the NP size. Thus, the NPs prepared in this work retain their ability to show positive contrast enhancement at larger particle sizes. This feature is favorable when large MRI–NIR bimodal probes are required. Notably, the fluorescence intensity of the RED-CNPs decreases remarkably with a decrease in the NP size [67]. Therefore, it is necessary to explore the optimum particle size for balancing the ability of the NP to facilitate effective *T*_1_-weighted contrast and fluorescence imaging at the same time.

### 3.3. In vivo MRI of a mouse body using PEG-NaGdF_4_:Yb^3+^,Er^3+^ NPs

**Figure 4.**
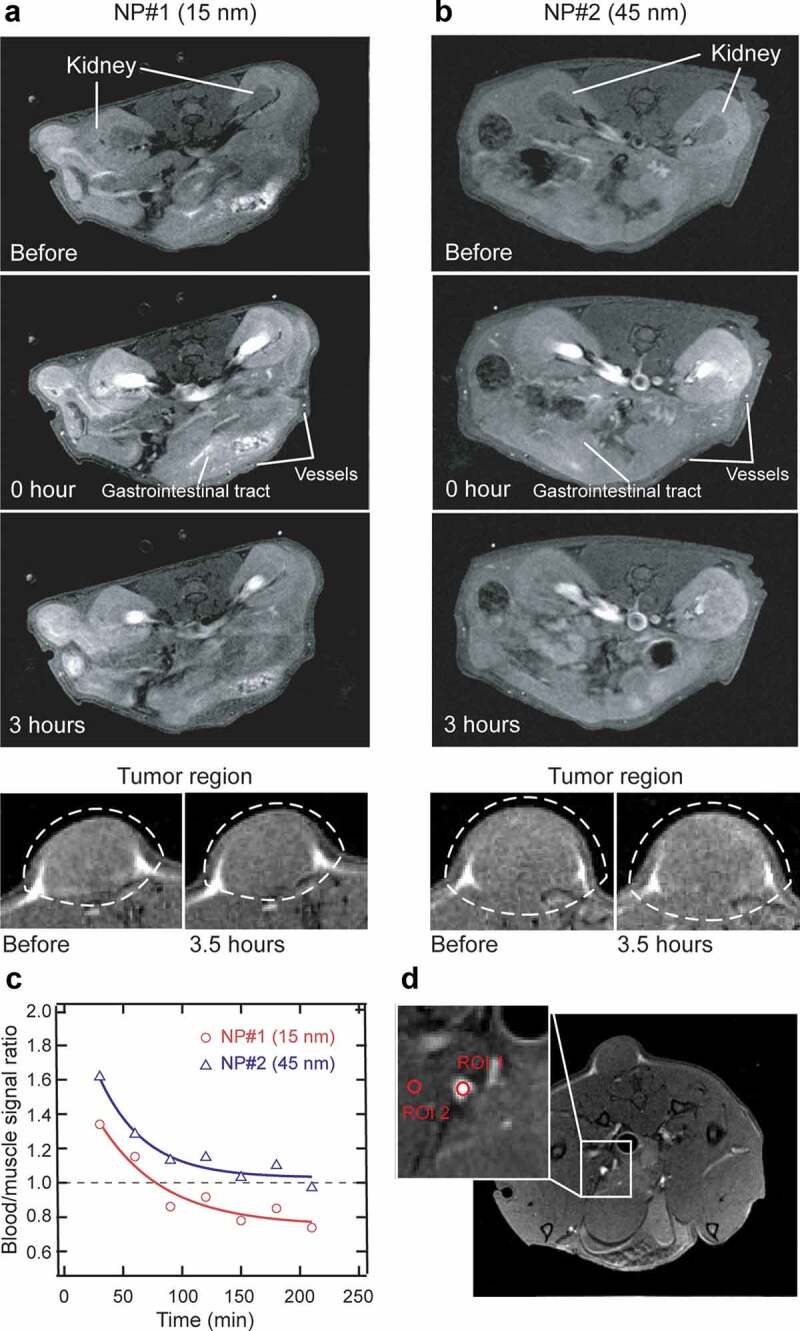
*In vivo* MRI results of Colon-26 subcutaneous tumor-bearing Balb/c nude mice following the tail-vein injection of PEGylated NaGdF_4_:Yb^3+^,Er^3+^ NPs as contrast agents. Representative transaxial slice of the *T*_1_-weighted images of the Balb/c nude mouse body (upper) and around the tumor site (bottom) contrasted by (a) 15 nm PEG-NaGdF_4_:Yb^3+^,Er^3+^ NPs and (b) 45 nm PEG-NaGdF_4_:Yb^3+^,Er^3+^ NPs. The kidney, blood vessels, and gastrointestinal tract are indicated by white arrows. (c) Blood-to-muscle contrast ratio at each time point, up to 210 min after the NP injection. (d) Transverse MR image of the tumor-bearing mouse used for calculating the blood-to-muscle contrast ratio. Regions of interest (ROI) for the blood and muscle are indicated by red circles as ROI 1 and ROI 2, respectively

The suspensions of 15 and 45 nm PEG-NaGdF_4_ NPs (NP#1 and NP#2, respectively) in saline water were used for *in vivo* MRI. Figure 4(a,b) show the *T*_1_-weighted images of the transaxial slice of the mouse body (upper) and the region around the tumor site (bottom) obtained before and immediately after the administration of NP#1 and NP#2 into the tumor-bearing mouse and after 3.5 h following the NP injection, as obtained with a 1.0 T MRI scanner. Compared with the images obtained before the NP injection, the images of the renal pelvis, renal cortex, pancreas, blood vessels, and gastrointestinal tract are clearly enhanced after the administration of both NP#1 and NP#2. Horizontal slices that feature the pancreas are shown in Figure S3. The signal enhancement due to the accumulation of the NPs on the surface of the tumor is not clearly observed after 3.5 h of the NP administration because the NPs are not designed for tumor targeting. The visualization of the blood vessels was enhanced significantly both by NP#1 and NP#2, and the visualization was better with NP#2 than with NP#1 because initial blood-to-muscle contrast ratio of NP#2 was 20% higher than that of NP#1 (Figure 4(c)). The blood-to-muscle contrast ratio was normalized with the blood signal obtained immediately after the injection, and ROI used for blood and muscle signals are displayed in Figure 4(d). Both NP#1 and NP#2 showed a similar half-life in blood (approximately 60–120 min). Nevertheless, NP#2 facilitated a relatively better blood vessel enhancement than NP#1 did, possibly due to its passive accumulation in the tumor region through the enhanced permeability and retention effect owing to endothelial leakages in the tumor neovascularization and the reduced lymphatic circulation in the tumor microenvironment [68]. In terms of safety, a long blood half-life of the NPs is potentially risky owing to the possibility of the permanent accumulation of Gd^3+^ and systemic toxicity, which can cause conditions, such as fibrosis. Therefore, the relatively rapid clearance of the PEG-NaGdF_4_:Yb^3+^,Er^3+^ NPs is beneficial for ensuring safety in future clinical applications.

### 3.4. In vivo OTN-NIR fluorescence imaging of mouse blood vessels

**Figure 5.**
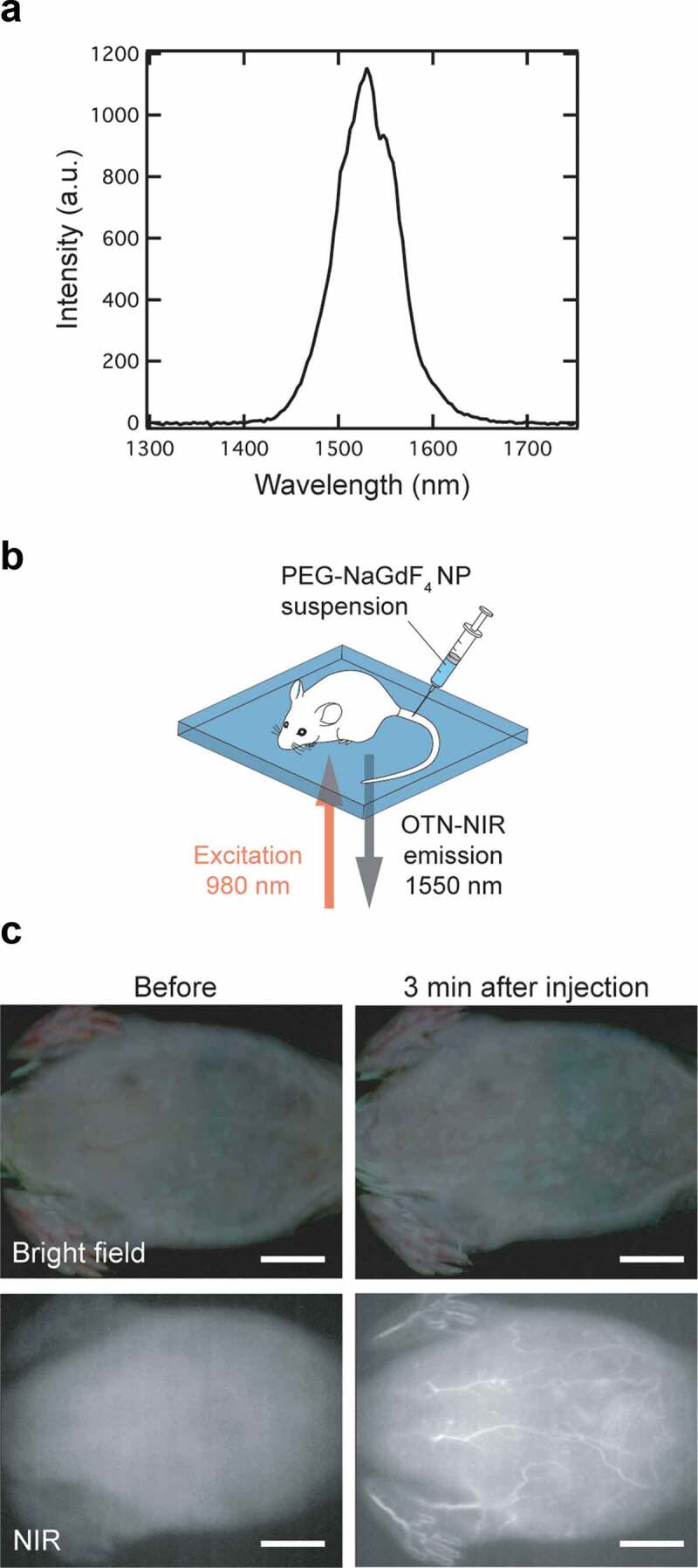
NIR fluorescence imaging of a live mouse. (a) OTN-NIR down-conversion fluorescence spectrum of PEG-NaGdF_4_:Yb^3+^,Er^3+^ NPs (Spectral acquisition conditions: dispersion medium, distilled water; excitation wavelength, 980 nm; power, 0.5 mW/cm^2^). (b) Illustration of the NIR fluorescence imaging of a live mouse under 980 nm excitation (0.5 W/cm^2^) using an SAI-1000 imaging system. (c) NIR fluorescence images (lower) and images under bright-field illumination (upper) of a live mouse before (left) and after 3 min (right) of the administration of NP#1. Scale bars represent 10 mm

NaGdF_4_:Yb^3+^,Er^3+^ NPs show strong NIR emission, providing a high signal-to-noise ratio and deeper penetration of biological tissues. The 15 nm PEG-NaGdF_4_:Yb^3+^,Er^3+^ NPs (NP#1) exhibited a distinct NIR emission under 980 nm excitation (see Figure 5(a)). To investigate the applicability of the NPs in the OTN-NIR bioimaging, we performed *in vivo* OTN-NIR fluorescence imaging of live mice after the intravenous injection of the NP#1 suspension (containing 10 µmol of Gd^3+^); the NPs were dispersed in physiological saline before injection. The PEG-NaGdF_4_:Yb^3+^,Er^3+^ NPs were excited with a 980 nm laser diode (power density: 0.5 W/cm^2;^ exposure time: 500 ms) and imaged using an NIR fluorescence imager (SAI-1000); the abdominal images of the live mouse were acquired, as schematically shown in Figure 5(b). Figure 5(c) shows the OTN-NIR fluorescence images of the live ICR mouse before and 3 min after the injection of NP#1 via the tail vein under NIR excitation, along with the images acquired under bright-field illumination as references. Compared with the image obtained before injection, the blood vessels located in the abdominal muscle were observed more vividly after the injection of the PEG-NaGdF_4_:Yb^3+^,Er^3+^ NPs, demonstrating the potential of these NPs as OTN-NIR probes. These preliminary results demonstrate the feasibility of the practical application of our size-controlled NPs for visualizing the blood vessels through MRI and NIR fluorescence imaging.

## 4. Conclusions

We demonstrated bimodal imaging contrast agents based on monodisperse β-NaGdF_4_:Yb^3+^,Er^3+^ NPs that are applicable to OTN-NIR fluorescence imaging and *T*_1_-weighted MRI. The size of the PEG-NaGdF_4_:Yb^3+^,Er^3+^ NPs was precisely controlled using a stepwise crystal growth method that allows the facile tuning of the thickness of the RED-crystalline layer, and NPs with hydrodynamic diameters of 15 and 45 nm were obtained. This was realized by controlling the number density of the seed NPs added to the reaction mixture. MRI *T*_1_ and *T*_2_ relaxivity measurements on the PEG-NaGdF_4_Yb^3+^,Er^3+^ NPs revealed a higher *r*_1_ than that of the clinical standard, Gd-DOTA, and the highest *r*_1_/*r*_2_ ratio, which defines the ability of ‘positive’ *T*_1_ contrast agents to enhance image contrast, was reported for NaGdF_4_-based NPs. The cell viability test confirmed that the NPs have low toxicity to NIH3T3 cells at <1 mM Gd^3+^ concentration. *In vivo* imaging of the blood vessels of live mice was achieved both by OTN-NIR fluorescence imaging and by MRI with the intravenous administration of the PEG-NaGdF_4_:Yb^3+^,Er^3+^ NPs. A short half-life of the NPs in the blood can ensure safety when applied clinically. For developing OTN-NIR/MRI bimodal contrast agents in such a size range, a precise synthesis for controlling their size is critical in terms of realizing their optimal performance as an MRI contrast agent as well as a fluorescence probe and for regulating their function in the body.

## Supplementary Material

Supplemental Material
