# Supplementary material for: Size-controlled bimodal in vivo nanoprobes as near-infrared phosphors and positive contrast agents for magnetic resonance imaging
Source: Sci Technol Adv Mater. 2021 Mar 10;22(1):160–72. doi: 10.1080/14686996.2021.1887712 (PMC7952065; doi:10.1080/14686996.2021.1887712)
Supplement: Supplemental Material [file TSTA_A_1887712_SM9639.docx]

**Size-controlled bimodal *in vivo* nanoprobes as near-infrared phosphors and positive contrast agents for magnetic resonance imaging**

Kyohei Okubo^a,*^, Ryuta Takeda^a^, Shuhei Murayama^b^, Masakazu Umezawa^a^, Masao Kamimura^a^, Kensuke Osada^b^, Ichio Aoki^b^, and Kohei Soga^a^

*^a^Department of Materials Science and Technology, Tokyo University of Science, Tokyo, Japan; ^b^National Institutes for Quantum and Radiological Science and Technology (QST), Chiba, Japan*

^*^kyohei.okubo@rs.tus.ac.jp


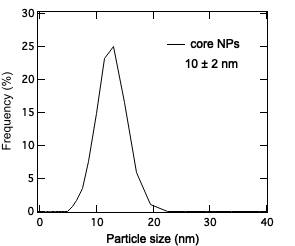


Figure S1. Particle size distribution of the OA-capped core NPs. Methodology: A DLS particle size analyser (LB-250, Horiba, Japan) was used for measuring the size distribution of OA-capped core NaGdF_4_:Yb^3+^,Er^3+^ NPs dispersed in cyclohexane with 1.0 mg/mL of sample concentration.


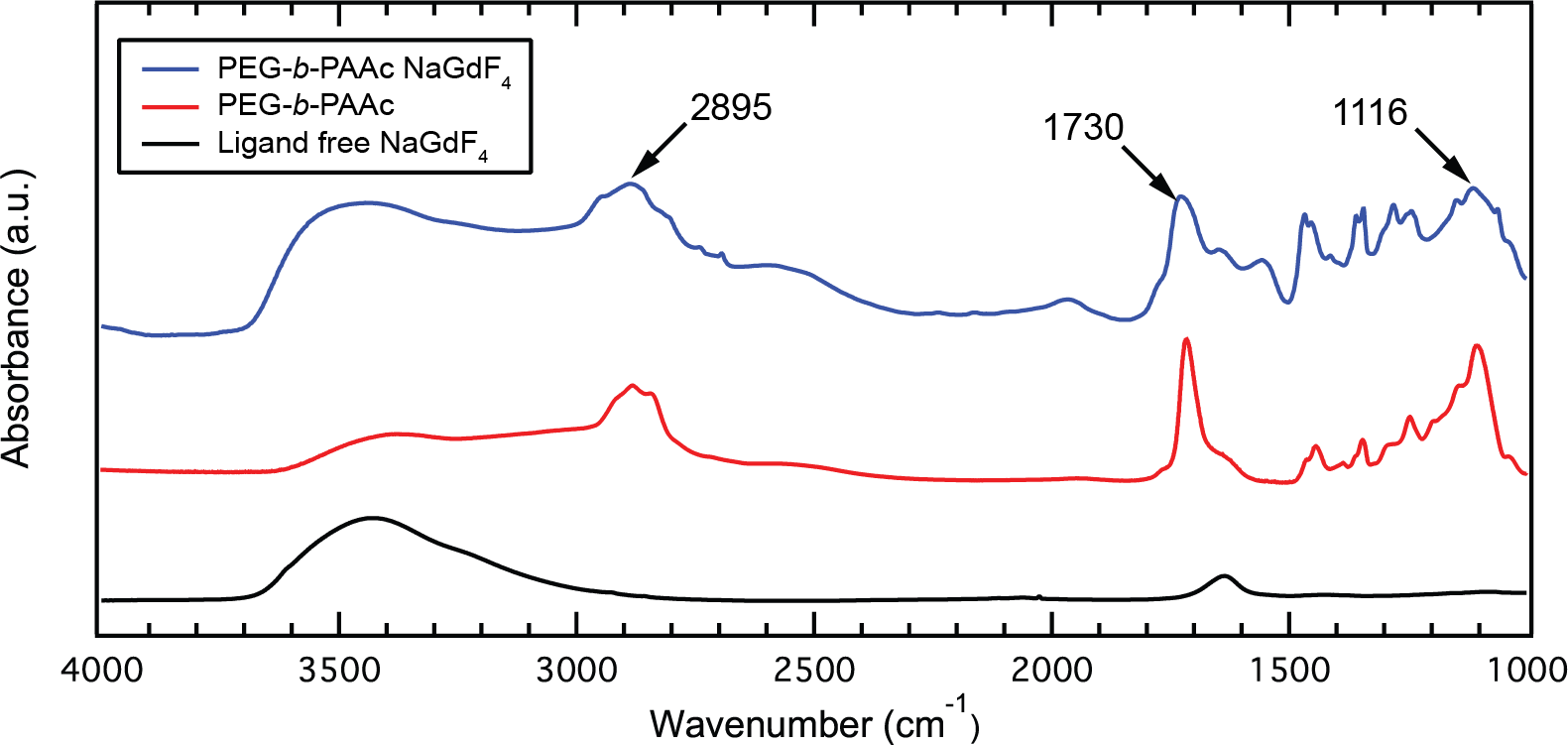


Figure S2. Fourier-transformed infrared (FTIR) absorption spectra of NaGdF_4_:Yb^3+^,Er^3+^ NPs (NP#1) before and after PEGylation. Methodology: A FTIR spectrometer (FT/IR-6500, JASCO, Tokyo, Japan) was used for measuring the FTIR absorption spectra. PEG-*b*-PAAc modified NaGdF_4_:Yb^3+^,Er^3+^ NPs, ligand-free NaGdF_4_:Yb^3+^,Er^3+^ NPs, and PEG-*b*-PAAc were analyzed in KBr powder.


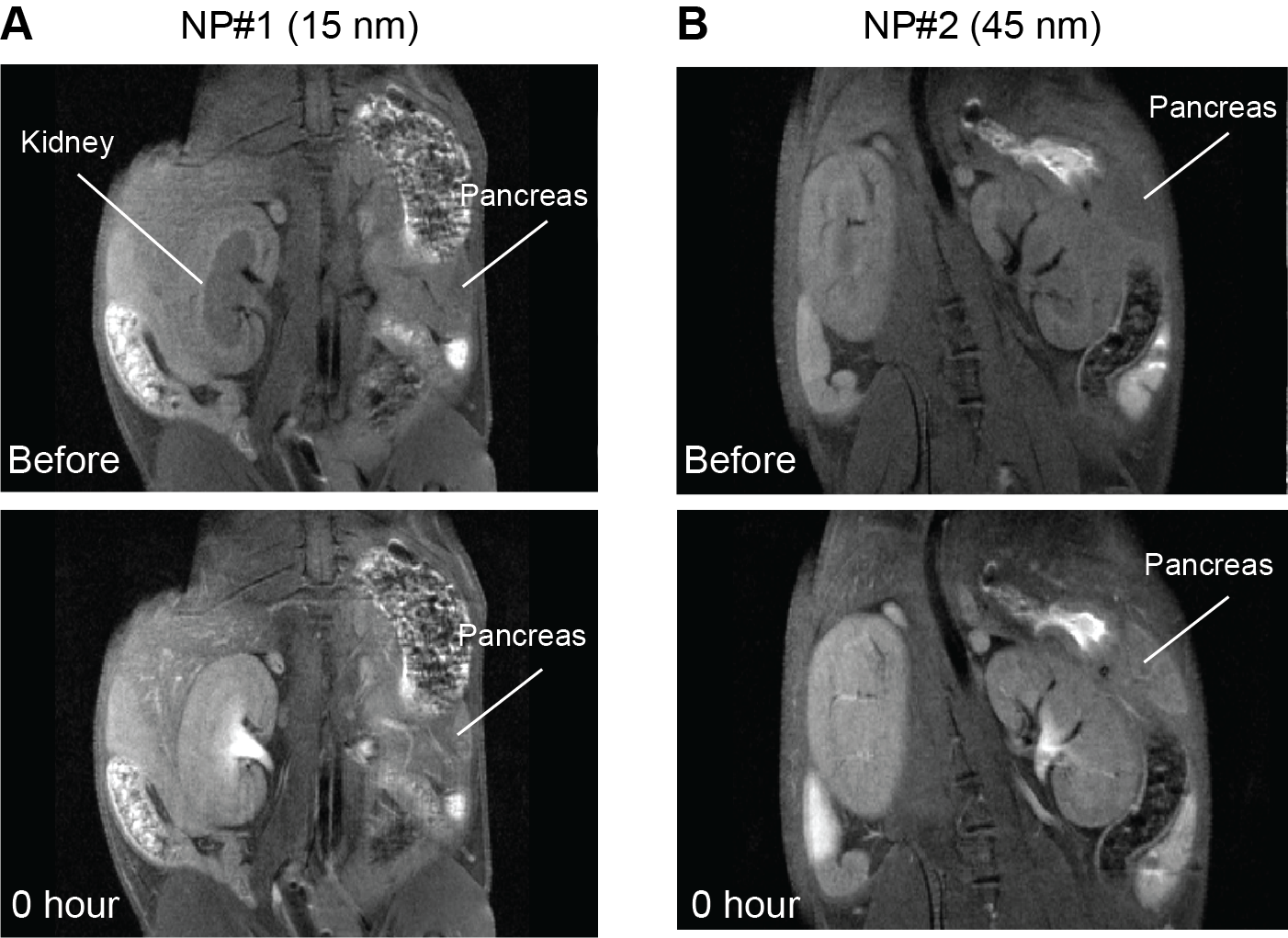


Figure S3. Representative horizontal slice of the *T*_1_-weighted images of the Balb/c nude mouse body before (upper) and after (lower) the tail-vain injection of (A) 15 nm PEGylated NaGdF_4_:Yb^3+^,Er^3+^ NPs (NP#1) and (B) 45 nm PEGylated NaGdF_4_:Yb^3+^,Er^3+^ NPs (NP#2), respectively. The pancreas and kidney are indicated by white arrows.
